# Ex vivo NMR metabolomics approach using cerebrospinal fluid for the diagnosis of primary CNS lymphoma: Correlation with MR imaging characteristics

**DOI:** 10.1002/cam4.5083

**Published:** 2022-08-08

**Authors:** Jae Hyun Kim, Yong Jin An, Tae Min Kim, Jeong Eun Kim, Sunghyouk Park, Seung Hong Choi

**Affiliations:** ^1^ Department of Radiology and Institute of Radiation Medicine Seoul National University Hospital Seoul Republic of Korea; ^2^ College of Pharmacy, Natural Product Research Institute Seoul National University Seoul Republic of Korea; ^3^ Department of Internal Medicine Seoul National University Hospital Seoul Republic of Korea; ^4^ Department of Neurosurgery Seoul National University Hospital Seoul Republic of Korea; ^5^ Center for Nanoparticle Research, Institute for Basic Science, and School of Chemical and Biological Engineering Seoul National University Seoul Republic of Korea

**Keywords:** apparent diffusion coefficient, cerebrospinal fluid, diagnosis, nuclear magnetic resonance, primary central nervous system lymphoma

## Abstract

**Purpose:**

Primary central nervous system lymphoma (PCNSL) is an uncommon extranodal non‐Hodgkin's lymphoma. Here, the feasibility of nuclear magnetic resonance (NMR) metabolomics for the diagnosis and prognosis prediction of PCNSL, as well as its correlation with magnetic resonance imaging (MRI) characteristics, was assessed.

**Patients and Methods:**

Cerebrospinal fluid (CSF) samples from PCNSL and normal groups (*n* = 41 for each) were obtained along with MRI data including pre‐ and postcontrast as well as T1‐, T2‐, and diffusion‐weighted imaging for the treatment‐naïve PCNSL patients (*n* = 24). The CSF samples were analyzed using nuclear magnetic resonance (NMR).

**Results:**

The CSF NMR metabolomic exhibited clear differences with a diagnostic sensitivity of 100% and a specificity of 97.6%. The citrate level of the leptomeningeal enhancement (LE) (+) group was significantly lower than that of the LE (−) group (*p* = 0.018). In addition, the MRI apparent diffusion coefficient (ADC) value of the tumor was positively correlated with the glucose level (*p* = 0.025). However, none of the marker metabolites were significant prognosis predictors in univariate analysis.

**Conclusions:**

In conclusion, the NMR metabolomics could be helpful to diagnose PCNSL, but not for the prognosis, and MRI features (LE or ADC) can reflect the metabolic profiles of PCNSL.

## Graphical abstract

For the treatment and prognosis of primary central nervous system lymphoma (PCNSL), an early and precise diagnosis is critical, but brain biopsy and cerebrospinal fluid (CSF) cytology are invasive and has poor diagnostic accuracy, respectively. In our study, NMR‐based analysis of CSF in PCNSL patients revealed a different metabolic profile. This is significantly correlated with magnetic resonance imaging characteristics. This approach could reduce costs, avoid invasive diagnostic procedures, and allow treatment to begin immediately for PCNSL patients.

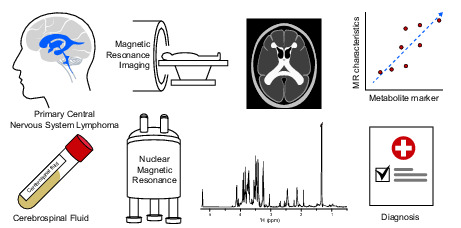

## 1 INTRODUCTION

Primary central nervous system lymphoma (PCNSL) is an aggressive primary brain cancer with rising incidence, especially in immunocompetent patients.^1^ For the attenuation of disease progression and neurologic deterioration, early and accurate diagnosis of PCNSL and the initiation of treatment are important^2^, ^3^ because unlike all other primary brain tumors, the prognosis of PCNSL does not improve with surgical resection,^4^ and definitive diagnosis by using a stereotactic brain biopsy is often necessary before starting treatment. Brain biopsy is, however, associated with a 1.2%–7% risk of hemorrhage and a 10%–35% risk of failure to achieve a histopathologic diagnosis.^5^ Furthermore, many patients are poor candidates for biopsy because of the tumor location in deep brain structures and patient‐related factors such as age and comorbidity. Cytologic examination of cerebrospinal fluid (CSF) is less invasive than brain biopsy, and CSF evaluation is needed for assessing PCNSL or the stage of non‐Hodgkin's lymphoma. However, cytologic examination of CSF only 2%–32% sensitivity in the diagnosis of CNS lymphoma.^6^, ^7^, ^8^ In addition, imaging modalities such as computed tomography (CT) and magnetic resonance imaging (MRI) are useful tools for the detection and differential diagnosis of brain lesions. Although PCNSL has characteristic imaging features on CT and MRI,^9^, ^10^, ^11^ none of these imaging features definitely differentiates PCNSL from other brain lesions. Therefore, it is crucial to develop a novel method for the diagnosis of PCNSL that is less invasive than brain biopsy and more accurate than CSF cytology or imaging modalities.

Metabolomics is a promising field in cancer research on the comprehensive profiling of metabolites in a sample such as cancer tissue or biofluids from cancer patients that can reflect altered metabolic pathways in cancer. Metabolic profiling based on nuclear magnetic resonance (NMR) can be used to investigate multiple metabolic changes simultaneously in pathological processes and characterize the dynamic metabolic responses of key intermediary biochemical pathways.^12^ Furthermore, many types of samples such as serum,^13^ urine,^14^ breath,^15^ and CSF^16^, ^17^ have been used in metabolomics studies of cancer, and these studies have attempted to find novel biomarkers for the early detection of various cancers. With the development of metabolomics technology within the past few years, remarkable improvements have been achieved in cancer diagnosis and prognosis based on the metabolomics approach. To our knowledge, however, there have been no clinical studies that have evaluated the usefulness of a metabolomics approach in the diagnosis of PCNSL.

Therefore, this prospective study was conducted to assess the feasibility of ex vivo NMR metabolomics for the diagnosis of PCNSL as well as the correlation between the metabolomics profile and MRI characteristics.

## 2 MATERIALS AND METHODS

More information on materials and methods is available in supplementary material.

### 2.1 Patients and sample collection

Approval for this study was obtained from the institutional review board of our hospital. All enrolled patients provided written informed consent.

Between January 2012 and January 2014, we enrolled immunocompetent patients with PCNSL. Inclusion criteria were as follows: the patient (a) had a histopathologic diagnosis of PCNSL; (b) underwent a contrast‐enhanced brain MRI; (c) also underwent whole‐body positron emission tomography (PET) and a bone marrow biopsy for the exclusion of systemic lymphoma; and (d) were checked for intraocular spread by slit lamp examination. Patients with prior intrathecal chemotherapy, age < 18 years, CNS infection or traumatic CSF collection were excluded. Finally, 41 patients with PCNSL (27 male, 14 female; age range, 24–80 years; mean age, 60 years) were enrolled in the present study. Among the 41 patients with PCNSL, 24 patients (18 male, 6 female; age range, 39–80 years; mean age, 63 years) had no previous treatment and 17 patients (9 male, 8 female; age range, 24–68 years; mean age, 56 years) underwent previous treatment including systemic chemotherapy at the time of enrolment. Among the 24 patients without previous treatment, 20 patients had follow‐up MRI and clinical information after standard treatment. When we analyzed the correlation between the metabolomics profile and MRI characteristics, 24 patients without previous treatments were included because previous treatment such as chemotherapy could affect the MRI features of tumors. In addition, when we investigated the prognostic value of the metabolomics profile, we only included 20 patients who had no previous treatment and had follow‐up MRI and clinical information.

Over the identical period, 41 patients (11 male, 30 female; age range, 43–76 years; mean age, 63 years) underwent elective frontoparietal craniotomy for clipping an unruptured cerebral aneurysm, and in each patient, 1 ml of CSF was obtained immediately after dural incision, with blood contamination checked by visual inspection. The CSF samples were used as the normal group. None of the patients had any medical history of malignancy.

The CSF samples from patients with PCNSL and normal patients were obtained under fluoroscopy guidance^18^ and during operation, respectively. The samples were snap frozen under liquid nitrogen and kept at −80°C until the NMR analysis.

## 3 RESULTS

### 3.1 Patient characteristics

The clinical characteristics of the PCNSL and normal groups are presented in Table 1. A total of 41 patients each were enrolled in the PCNSL and normal groups, respectively. For the PCNSL group, the mean age was 60 years and 66% (27/41) were men; for the normal group, the mean age was 63 years and 27% (11/41) were men. The CSF protein level in the PCNSL group was higher than that in the normal group (mean ± standard deviation; 105 ± 73.6 vs. 35.6 ± 11.3). Of the 41 patients with PCNSL, 35 patients showed an elevated protein level (>45 mg/dl). The CSF from the normal group showed normal biochemical profiles.

**TABLE 1:** Patient characteristics

	PCNSL	Normal control	*p* value
Number of patients	41	41	
Age^a^	60.17 ± 10.75	62.71 ± 8.40	0.024
Gender			0.0009
Male	27	11	
Female	14	30	
Biochemical profile of CSF			
Protein (mg/dl)^a^	105.0 ± 73.6	35.6 ± 11.3	<0.0001

*Note*: Unless otherwise indicated, data are the number of patients.

Abbreviations: CSF, Cerebrospinal fluid; PCNSL, primary central nervous system lymphoma.

^a^ Data are the mean ± standard deviation.

### 3.2 NMR spectra of CSF from normal and PCNSL patients and multivariate analysis

**FIGURE 1:**
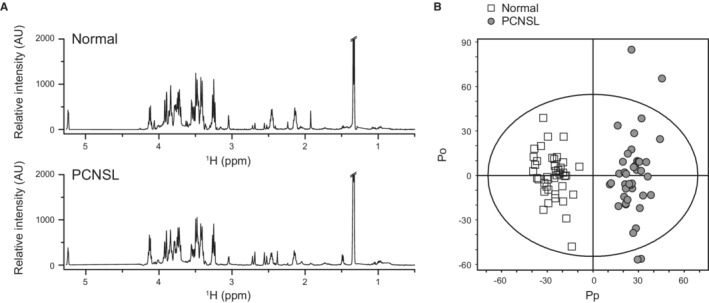
Averaged NMR spectra and multivariate analysis for the CSF obtained from normal and PCNSL patient groups. (A) The spectra were acquired for CSF of PCNLS samples on a 500 MHz ^1^H‐NMR spectrometer. (B) The OPLS‐DA model was obtained with one predictive (Pp) and two orthogonal components (Po). Open squares represent normal samples, and gray‐filled circles represent PCNSL samples. AU, arbitrary unit; CSF, cerebrospinal fluid; NMR, nuclear magnetic resonance; OPLS‐DA, orthogonal projections to latent structure‐discriminant analysis; PCNSL, primary central nervous system lymphoma.

One‐dimensional NMR spectra of the CSF samples from the 41 normal and 41 PCNSL patients were acquired for the possible application of CSF in the diagnosis of PCNSL. We then averaged all the spectra in each group to observe any differences in peak profiles according to lymphoma status (Figure 1A). Despite the overall similarity of the average spectra, there were noticeable differences, especially in the 1.5–3 ppm regions. We then identified metabolites corresponding to each peak based on coupling constants and chemical shifts (Table S1). As these average profiles suggested the existence of possible metabolic differences, multivariate statistical analysis was performed to analyze the data more holistically and to address intragroup variations more efficiently. A partial least squares‐discriminant analysis (PLS‐DA) model also showed good separation, goodness of fit of 0.955, and predictive values of 0.928 (Figure S1A). The statistical significance was also tested with permutation, which showed the validity of the analysis with proper decreases in R2 and Q2 values upon permutation (Figure S1B). An orthogonal projection to latent structure‐discriminant analysis (OPLS‐DA) model exhibited good separation between the two groups with a goodness of fit of 0.926 and predictive values of 0.899 (Figures 1B and S1C). Other statistical analyses also gave consistent results. Random forest (RF) classification showed good performance after 150 trees (Figures S2A and S2B). Support vector machine (SVM) classification showed a good classification with a 0% 10‐fold cross‐validation error rate (Figure S2C). Overall, these results suggest that the presence of lymphoma affects the metabolic profile of CSF, which may be exploited to differentiate normal and PCNSL groups.

### 3.3 PCNSL diagnosis using the metabolomics multivariate model

**FIGURE 2:**
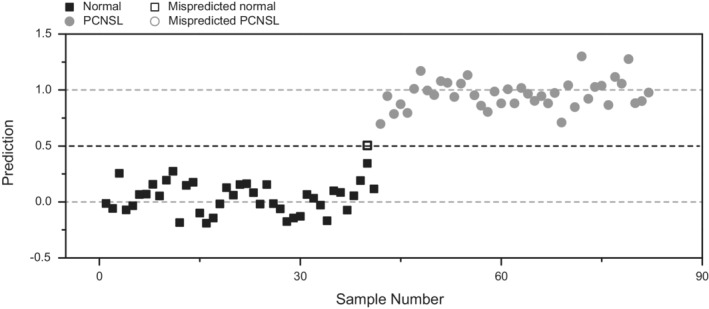
Leave‐one‐out cross‐validation with an OPLS‐DA model. The boxes represent normal samples, whereas the circles represent PCNSL samples. The filled symbols were obtained from the OPLS‐DA using the entire data set. The open symbols were mispredicted samples. The prediction result was determined by using the a priori value of 0.5 for the *Y* variable from the OPLS‐DA model. OPLS‐DA, orthogonal projections to latent structure‐discriminant analysis; PCNSL, primary central nervous system lymphoma.

As the multivariate OPLS‐DA model exhibited high statistical significance in distinguishing normal and PCNSL groups, it was tested for its ability to predict the lymphoma status of unknown samples. To that end, we performed a leave‐one‐out cross‐validation test of the obtained OPLS‐DA model, leaving out one sample at a time as an unknown. Using a priori cutoff value of 0.5, this cross‐validation correctly predicted the lymphoma status of 40 out of the 41 normal samples (specificity of 97.6%) and 41 out of the 41 PCNSL samples (a sensitivity of 100%) (Figure 2). RF model also exhibited good classification with a 0–0.0122 of out‐of‐bag error rate (Table S2). In addition, the significant features were also very similar to those from the OPLS‐DA model (Figures S1C and S2B).

### 3.4 Metabolites contributing to the differentiation

**FIGURE 3:**
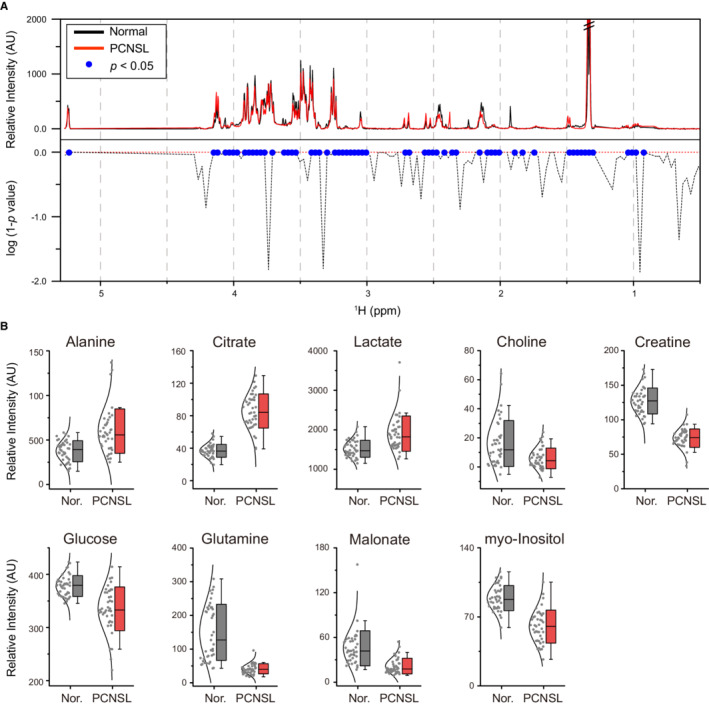
Statistical significance of the markers. (A) The upper spectrum shows the average NMR spectra for normal (black) and PCNSL (red) groups. The lower shows the Student's *t* test results of the entire NMR spectra. The X‐axis represents the ppm, and the Y‐axis represents the relative intensity (upper) as well as the log of the 1‐*p* values (lower). For the lower, blue dots represent the values with *p* < 0.05, and the red dot line represents the value of zero. (B) The bar charts show the relative intensities of the nine biomarkers for each sample, normal (Nor.) and PCNSL. AU, arbitrary unit; NMR, nuclear magnetic resonance; PCNSL, primary central nervous system lymphoma.

The diagnosis itself does not require the identification of individual metabolites contributing to the differentiation,^19^, ^20^ and, in fact, the diagnostic reliability should be better when using all the contributing signals at once. Nevertheless, the identification of differentially existing metabolites may help in understanding the underlying physiological changes of diseases or the development of simpler diagnostic approaches, such as kits. Therefore, we performed Student's *t* tests over all the metabolic signals in the NMR spectra and visualized the significance with the log of the 1 ‐ *p* values (Figure 3A). Matching the significantly different signal regions with the identified metabolites (see Table S1), we found possible marker metabolites for PCNSL. These include increases in alanine, citrate, and lactate in PCNSL CSF samples as well as decreases in choline, creatine, glucose, glutamine, malonate, and myo‐inositol in PCNSL CSF samples. All these metabolites exhibit very low *p* values (from 4.05 × 10^−4^ for choline down to 8.27 × 10^−24^ for creatine), despite comparatively smaller changes in the actual values (from −72% for glutamine up to 133% for citrate) (Figure 3B and Table S1). These results suggest that the combination of these nine metabolites should be the most significant feature differentiating the PLNSL and normal groups.

### 3.5 Correlation of marker metabolites with CSF protein level and MRI characteristics

**FIGURE 4:**
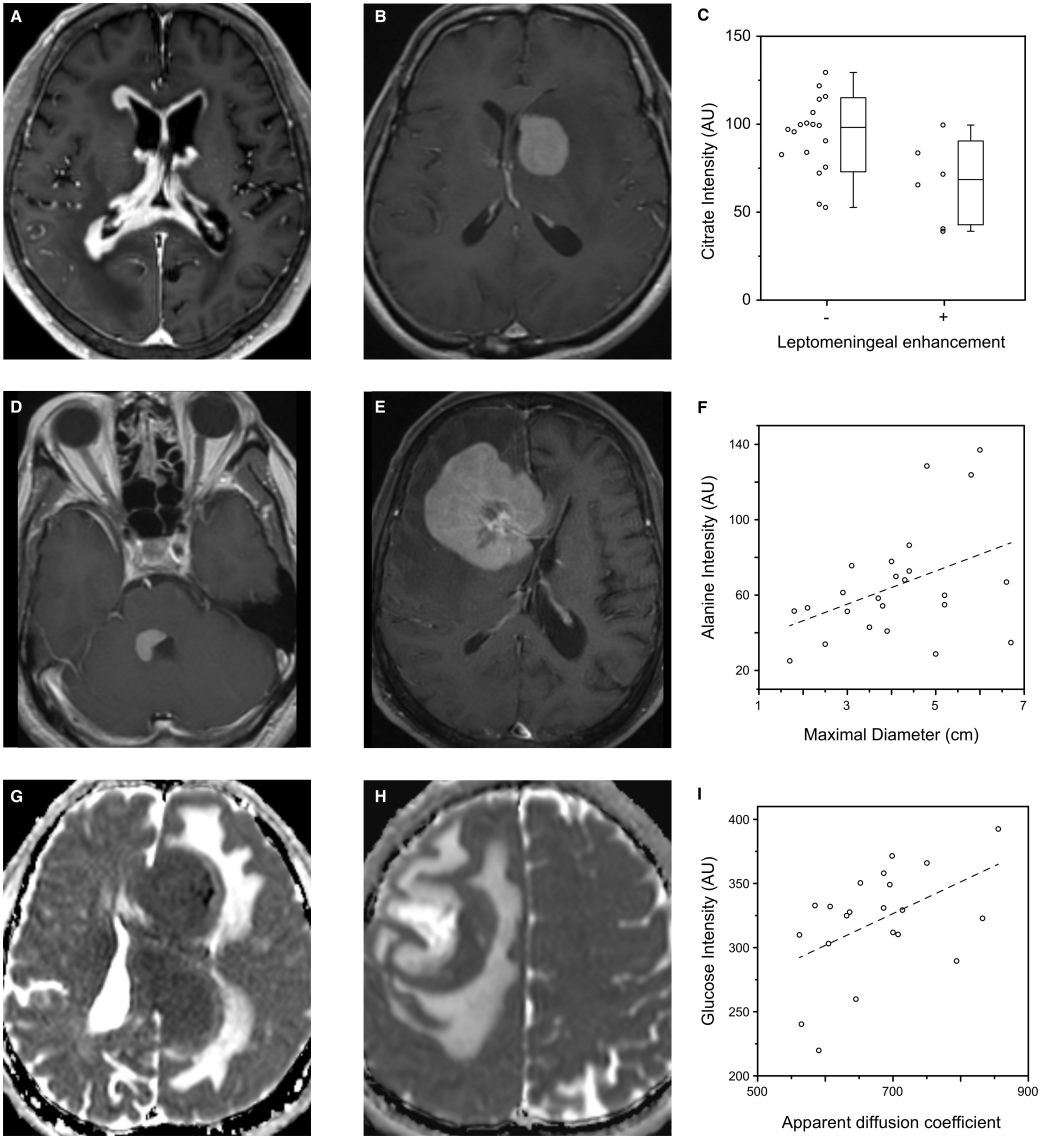
Correlation between marker metabolites and MR characteristics. The axial postcontrast T1‐weighted images show prominent ependymal enhancement in a 71‐year‐old male patient with a low citrate level (A) and a homogeneously enhanced mass in the left basal ganglia without evidence of leptomeningeal or ependymal enhancement in a 54‐year‐old male patient with a high citrate level (B). The bar plots of the citrate levels against the leptomeningeal enhancement of the tumor (*p* = 0.014) (C). A 62‐year‐old male patient with a low alanine level has a solitary enhancing lesion with a maximum diameter (1.6 cm) in the right cerebellar peduncle on the axial postcontrast T1‐weighted image (D). A 63‐year‐old male patient with a high alanine level has an enhancing lesion with a maximum diameter (5.8 cm) in the right frontal lobe on an axial postcontrast T1‐weighted image (E). The linear regression plots of the alanine levels against the maximum diameter of the tumor on a contrast‐enhanced T1‐weighted image (*R* ^2^ = 0.174, *p* = 0.043) (F). The apparent diffusion coefficient (ADC) maps show masses with low ADC values (561.8) in the left cerebral hemisphere in a 58‐year‐old male patient with a low glucose level (G) and a mass with high ADC values (750.2) in the right cerebral hemisphere in a 39‐year‐old male patient with a high glucose level (H). The linear regression plots of the glucose levels against the ADC values of the tumor (*R* ^2^ = 0.237, *p* = 0.025) (I).

With the identification of marker metabolites, we tested if they can be correlated with conventional parameters or modalities used in the diagnosis of PCSNL. First, we looked at the CSF protein level, as it was significantly different between normal and PCSNL groups (Table 1). There was statistically significant positive correlation between the CSF protein level and three marker metabolite levels (lactate, *p* = 0.046, *R* ^2^ = 0.169; malonate, *p* = 0.020, *R* ^2^ = 0.220; choline, *p* = 0.034, *R* ^2^ = 0.189). In addition, the CSF protein level was negatively correlated with two metabolite levels (citrate, *p* = 0.026, *R* ^2^ = 0.205; glucose, *p* = 0.021, *R* ^2^ = 0.220). In comparison, there were no significant correlations between the CSF protein level and the other marker metabolites (alanine, glutamine, creatine, and myo‐inositol). Multiple linear regression analysis by using nine marker metabolites showed that malonate (*β* = 3.787, *p* = 0.048), citrate (*β* = −0.777, *p* = 0.023), and glucose (*β* = −1.317, *p* = 0.024) were variables that had a statistically significant independent correlation with the CSF protein level. Second, MRI characteristics were also tested, as it is an important diagnostic modality. The citrate level of the leptomeningeal enhancement (+) group was significantly lower than that of the leptomeningeal enhancement (−) group (median; 68.5 vs. 98.2; *p* = 0.018) (Figures 4A–C). Additionally, the maximum diameter of the tumor on contrast‐enhanced T1WI was positively correlated with the alanine level (*p* = 0.043, *R* ^2^ = 0.174) (Figures 4D–F). Furthermore, there was a significant positive correlation between the ADC value of the tumor and the glucose level (*p* = 0.025, *R* ^2^ = 0.237) (Figures 4G–I). There were no significant differences in marker metabolite levels according to other MRI characteristics including lesion multiplicity and contact with the CSF space.

### 3.6 Correlation between progression‐free survival and marker metabolites

As we obtained excellent results from metabolomics analysis for the diagnosis of PCNSL and related parameters, and we also tested if the analysis can be correlated with the PCNSL prognosis in terms of progression‐free survival. Univariate analysis of marker metabolite levels as continuous variables by using the Cox model demonstrated that there were no significant predictors of progression (Table 2).

**TABLE 2:** Progression‐free survival with univariate cox proportional hazard analysis

Characteristic	No. of patients (*n* = 20)	*p* value	HR	95% CI
Age				
≤60	7	–	1.000	–
>60	13	0.570	0.690	0.192, 2.478
Eastern Cooperative Oncology Group (ECOG) performance status				
≤1	17	–	1.000	–
>1	3	0.042	5.925	1.069, 32.823
Serum LDH level				
Normal	12	–	1.000	–
High	8	0.133	0.304	0.064, 1.439
CSF protein level				
Normal	1	–	1.000	–
High	19	0.405	0.406	0.049, 3.383
Involvement of deep structures				
Negative	2	–	1.000	–
Positive	18	0.881	0.851	0.103, 7.027
Marker metabolites				
Citrate	20	0.747	1.005	0.976, 1.035
Lactate	20	0.179	1.001	1.000, 1.002
Myo‐inositol	20	0.133	0.969	0.931, 1.010
Creatine	20	0.126	0.968	0.928, 1.009
Glutamine	20	0.891	0.998	0.964, 1.032
Alanine	20	0.180	1.013	0.994, 1.032
Malonate	20	0.476	1.050	0.918, 1.201
Choline	20	0.087	0.809	0.634, 1.032
Glucose	20	0.367	0.991	0.972, 1.010

Abbreviations: CI, confidence interval; CSF, Cerebrospinal fluid; HR, hazard ratio; LDH, lactate dehydrogenase.

## 4 DISCUSSION

The aim of the present study was to assess the feasibility of ex vivo NMR metabolomics for the diagnosis and prognosis prediction of PCNSL as well as the correlation between the metabolomics profile and MRI characteristics. Our study results revealed that the NMR spectra of CSF were significantly different between the PCNSL and normal groups. Furthermore, the leave‐one‐out cross‐validation approach based on the NMR spectra showed excellent performance for the diagnosis of PCNSL, with a specificity of 97.6% and a sensitivity of 100%. Although the CSF protein level of the PCNSL group was also significantly higher than that of the normal group in baseline laboratory findings, the elevated CSF protein level is nonspecific and may result from other various conditions such as hemorrhage, inflammatory, demyelinating, and autoimmune diseases. In comparison, the sensitivity of CSF cytology is low and varies widely in CNS lymphoma with values ranging from 2% to 32%.^6^, ^7^, ^8^ In leptomeningeal carcinomatosis, the sensitivity of cytology is approximately 55% for the first CSF examination.^21^ Although CSF cytology shows a high specificity of ~100%, low sensitivity is a significant disadvantage for PCNSL diagnosis. In addition, contrast‐enhanced MRI is the method of choice for detecting PCNSL. Typical PCNSL shows hypo‐ or isointense on T1WI, iso‐ to hyperintense on T2WI, and homogeneous contrast enhancement on contrast‐enhanced T1WI.^10^, ^22^ However, none of these typical imaging features can unequivocally differentiate PCNSL from other brain lesions. Therefore, the NMR metabolomics approach may be combined with the conventional modalities for better PCNSL diagnosis. Of note, the NMR metabolomics approach has several merits as a diagnostic modality. First, the data can be obtained with a very small volume of CSF (0.5 cc). Second, the interpretation of the metabolic profile may be automated if desired and thus may not require expert pathologists or radiologists. Third, because NMR data acquisition costs 5–10 US dollars per sample in a common facility, the NMR metabolomics approach may be a cost‐effective diagnostic modality.

One of the important findings of our study is that the PCNSL group showed characteristic metabolomic markers such as increases in lactate, citrate, and alanine levels along with decreases in choline, creatine, glucose, glutamine, malonate, and myo‐inositol levels compared with those of the normal group. Lactate is widely known as a metabolic key player in cancer. The malignant transformation of normal cells leads to increased glucose uptake and lactate formation even under normoxic conditions in most solid tumors (i.e., aerobic glycolysis or the Warburg effect).^23^ Furthermore, several previous studies demonstrated that MR spectroscopy of PCNSL revealed an increased lactate signal.^24^, ^25^ The increased alanine level of PCNSL could be the result of the lactate increase because pyruvate produced by the Warburg effect is converted to alanine for regulating lactate production.^26^ Citrate also plays an important role in cancer cell metabolism. Mitochondrial citrate diffusing in the cytosol, which restores oxaloacetate and acetyl‐CoA, allows cancer cells to burn lipid and protein reserves in order to sustain their own biosynthetic pathways.^27^, ^28^ Although there was no mention of a citrate signal in previous MR spectroscopy studies of PCNSL, some studies demonstrated that gliomas showed increased citrate levels.^29^, ^30^ Myo‐inositol is found primarily in astrocytes and is a precursor for secondary messenger systems, functioning as a brain osmolyte.^31^, ^32^ In addition, some previous studies proved that the myo‐inositol/creatine ratio or creatine level has an inverse correlation with the glioma grade.^33^, ^34^ Decreased creatine and myo‐inositol levels of PCNSL in our study are consistent with the results of previous studies using MR spectroscopy.^25^, ^35^ Furthermore, decreased creatine levels were also found in glioblastoma and other various brain tumors.^36^, ^37^ It is well known that glucose is an essential fuel for most glycolysis‐dependent brain tumor cells and glucose elevation is associated with a poor prognosis.^38^, ^39^ Glutamine is also a major metabolic fuel for brain tumor cells, as it donates its nitrogen and carbon atoms into an array of growth‐promoting pathways.^40^, ^41^ In addition, several previous studies using MR spectroscopy suggested that increased choline might reflect increased cell turnover or cellularity of brain tumors.^42^ Our results, which showed decreased glucose, glutamine, and choline levels of PCNSL, seem to contradict these previous reports. Although we cannot explain the exact cause of the differences in those metabolite levels between PCNSL and other brain tumors, it could be a unique metabolomics profile that allows us to differentiate PCNSL from other brain tumors. Furthermore, because there have been no previous studies that have mentioned the malonate level of brain tumors, the decreased malonate level of PCNSL could also be a characteristic finding of the metabolomics profile of PCNSL. Therefore, these characteristic marker metabolites of PCNSL could provide helpful information for the accurate diagnosis of PCNSL.

We also found that three MRI characteristics were significantly correlated with marker metabolite levels. First, the citrate level of the leptomeningeal enhancement (+) group was significantly lower than that of the leptomeningeal enhancement (−) group (median; 68.2 vs. 97.9; *p* = 0.02). This finding indicates that the location of cancer cells could affect the metabolic profile of the CSF in PCNSL. However, since there is a lack of articles evaluating the correlation between CSF citrate and leptomeningeal cancer cells, we may not be able to provide an exact cause of the decrease in the citrate level in the leptomeningeal enhancement (+) group. We only speculated that leptomeningeal cancer cells might have different metabolic characteristics compared with cancer cells in brain parenchyma. Second, the maximum diameter of the tumor in contrast‐enhanced T1WI was positively correlated with the alanine level (*p* = 0.043, *R* ^2^ = 0.174). Several previous studies have proven that lactate and alanine levels of cancer are upregulated because of increased glycolysis and cell membrane biosynthesis.^43^, ^44^ In addition, in a previous study regarding a hepatocellular carcinoma mouse model, the metabolic flux of pyruvate to alanine significantly increased with tumor size.^45^ Therefore, the positive correlation between the tumor diameter of PCNSL and alanine level could be related to the enhanced conversion of pyruvate to alanine. Third, there was a significant positive correlation between the apparent diffusion coefficient (ADC) value of the tumor and glucose level (*p* = 0.025, *R* ^2^ = 0.237). Recent studies have shown an inverse correlation between the ADC and standardized uptake values (SUV) of ^18^F‐fluoro‐deoxy‐glucose‐positron emission tomography (^18^F‐FDG PET) in lymph nodes in lymphoma patients^46^ and brain tumors.^47^ The result of our study is the exact opposite to that of previous studies. However, we measured the glucose level of the CSF fluid instead of the tumor. Therefore, the decrease in the CSF glucose level in PCNSL could be caused by the consumption of glucose by the tumor cells.

In terms of the prediction of the prognosis of PCNSL, our study results showed that there are no significant predictors of progression among marker metabolites. However, several previous studies demonstrated that MR spectroscopy metabolites were significantly correlated to the prognosis of high‐grade glioma patients.^48^ Considering that well‐known prognostic factors of PCNSL such as age, serum level of lactate dehydrogenase, CSF protein concentration, and involvement of deep regions of the brain did not show statistical significance in the current study either, these results could be due to the small study population for the prognostic test (20 out of the total of 41).

Our study has several limitations. First, our study had a relatively small number of subjects, especially for the evaluation of prognosis predictability. It would be necessary to perform larger studies to validate our findings. Second, we did not study the differences in metabolomic profiles between PCNSL and other primary brain tumors. It would also be necessary to perform further studies to make the NMR metabolomics approach into a definitive diagnostic tool of PCNSL.

In conclusion, the NMR metabolomics approach might be a helpful modality for the diagnosis of PCNSL, and the assessment of MRI features such as the leptomeningeal enhancement, maximum tumor diameter on contrast‐enhanced T1WI, and ADC value can reflect the metabolic profiles of PCNSL. However, marker metabolites of PCNSL cannot be used for the prediction of the prognosis.

## AUTHOR CONTRIBUTIONS

Jae Hyun Kim: Formal analysis, Investigation, Writing—Original draft preparation; Yong Jin An: Formal analysis, Investigation, Writing—Original draft preparation; Tae Min Kim: Investigation; Jeong Eun Kim: Investigation; Sunghyouk Park: Conceptualization, Writing—Original draft preparation, Methodology; and Seung Hong Choi: Conceptualization, Writing—Original draft preparation, Methodology.

## CONFLICT OF INTEREST

The authors declare that they have no competing financial interests.

## Supporting information


Figure S1
Figure S2
Table S1
Table S2

## Data Availability

The data that support the findings of this study are available from the corresponding author upon reasonable request.
