# Supplementary material for: Ex vivo NMR metabolomics approach using cerebrospinal fluid for the diagnosis of primary CNS lymphoma: Correlation with MR imaging characteristics
Source: Cancer Med. 2022 Aug 8;12(4):4679–89. doi: 10.1002/cam4.5083 (PMC9972060; doi:10.1002/cam4.5083)
Supplement: Supplementary file 1 — Figure S1 Figure S2 Table S1 Table S2 [file CAM4-12-4679-s001.doc]

**Ex vivo NMR Metabolomics Approach using Cerebrospinal Fluid for the Diagnosis of Primary CNS Lymphoma: Correlation with MR Imaging Characteristics**

Jae Hyun Kim ^a, 1^, Yong Jin An ^b, 1^, Tae Min Kim ^c^, Jeong Eun Kim ^d^, Sunghyouk Park ^b, *^, Seung Hong Choi ^a, e, *^

^a^ Department of Radiology and Institute of Radiation Medicine, Seoul National University Hospital, 101, Daehak-ro Jongno-gu, 03080, Seoul, Republic of Korea

^b^ College of Pharmacy, Natural Product Research Institute, Seoul National University, San 56-1 Sillim-dong, Gwanak-gu, 08826, Seoul, Republic of Korea

^c^ Department of Internal Medicine, Seoul National University Hospital, 101, Daehak-ro Jongno-gu, 03080, Seoul, Korea Seoul, Republic of Korea

^d^ Department of Neurosurgery, Seoul National University Hospital, 101, Daehak-ro Jongno-gu, 03080, Seoul, Republic of Korea

^e^ Center for Nanoparticle Research, Institute for Basic Science, and School of Chemical and Biological Engineering, Seoul National University, San 56-1 Sillim-dong, Gwanak-gu, 08826, Seoul, Republic of Korea

**^1^ These authors contributed equally to this work**

*** Corresponding author**

Sunghyouk Park, College of Pharmacy, Seoul National University, San 56-1, Sillim-dong, Gwanak-gu, 08826, Seoul, Republic of Korea, Tel.: 182-2-880-7831, Fax: +82-2-880-0649, E-mail: psh@snu.ac.kr

Seung Hong Choi, Department of Radiology, Seoul National University Hospital, 101 Daehak-ro, Jongno-gu, 03080, Seoul, Republic of Korea, Tel.: +82-2-2072-2584, E-mail: verocay@snuh.org

**MATERIALS AND METHODS**

**Follow-up and Progression Assessment**

The 20 patients without previous treatment were followed up on during a median period of 14.8 months (range, 1.6-33.9 months). Clinical information and follow-up magnetic resonance imaging (MRI) were used to assess the patients. The determination of the progression of the disease was based on the standardized response criteria proposed by the International Primary CNS Lymphoma Collaborative Group (IPCG) [^1^](#_ENREF_1). The patients who met any one of following criteria were classified as having a progressive disease: (a) a more than 25% increase in the contrast enhancing lesion seen on MRI as compared with the baseline or best response (comparison should be made to the smallest of multiple lesions); (b) progression of ocular disease as indicated by an increase in the vitreous cell count or progressive retinal or optic nerve infiltration; or (c) appearance of any new lesion or site of disease (ocular, leptomeningeal or systemic) during or at the end of therapy. One radiologist (S.H.C.; 14 years of brain MRI experience) reviewed all follow-up MRI images obtained from the study population (n=20). In addition, the progression-free survival for patients was calculated from the time of entry into the study until disease progression or death as a result of PCNSL.

**Histological Subtype and Treatment Regimen**

The histological subtypes of 42 patients with PCNSL were diffuse large B-cell lymphoma (n=39), T-cell lymphoma (n=1), lymphomatosis cerebri (n=1), and unclassified (n=1). Regarding treatment regimen, all patients (n=42) underwent upfront high-dose methotrexate-based chemotherapy followed by whole-brain radiotherapy. Exact chemotherapy regimen was varied depending on the medical oncologist decision and treatment period. Twenty-one patients underwent combination chemotherapy with methotrexate, vincristine, and procarbazine.

**MR Techniques**

For each patient without previous treatment, MR imaging studies were performed using a 3-T scanner with a 32-channel head coil (n=9, Verio; Siemens Healthcare Sector, Erlangen, Germany) or a 1.5 T scanner (n=15, Signa HDxt; GE Medical Systems, Milwaukee, WI, USA). The MR imaging included T1-weighted image (T1WI) before and after contrast enhancement of multi-planar reconstructed transverse and coronal imaging with a sagittal three-dimensional magnetization prepared rapid acquisition gradient echo (3D-MPRAGE) sequence; transverse fluid-attenuated inversion recovery (FLAIR); transverse T2-weighted imaging (T2WI) with turbo spin-echo sequences; and diffusion-weighted imaging (DWI). We obtained the T1WI with 3D-MPRAGE sequences using the following parameters: Repetition Time (TR), 8.5-1500 ms; Echo Time (TE), 1.9-3.7 ms; Flip Angle (FA), 9-20°; matrix, 220-256 × 220-232; FOV, 220 × 220-250; section thickness, 1 mm; and Number of Excitation (NEX), 1. The parameters in axial FLAIR imaging were a TR of 8802-9,000 ms, a TE of 97-126.3 ms, a FA of 90-130°, a matrix of 320-384 × 192-348, a Field of view (FOV) of 199-220 × 220 and a slice thickness of 5 mm. The parameters in the transverse T2WI were as follows: TR, 4650-5160 ms; TE, 91-126.9 ms; FA, 90–130°; matrix, 448-640 × 256-580; FOV, 175-220 × 220; section thickness, 5 mm; and NEX, 2-3. Contrast-enhanced T1WI was obtained after the intravenous administration of Gadobutrol (Gadovist; Bayer Schering Pharma, Berlin, Germany) at a dose of 0.1 mmol/kg of body weight.

DWI was performed with a single-shot spin-echo EPI sequence in the axial plane before the injection of contrast material with a TR/TE of 6900-10050/55-75.4 ms at b = 0 and 1000 sec/mm^2^, 35-40 sections, a 3 mm section thickness, a 1 mm intersection gap, a FOV of 240 × 240 mm, a matrix of 160 × 160, three signal averages, and a voxel resolution of 1.5 × 1.5 × 3.0 mm. DWI was acquired in three orthogonal directions and combined into a trace image. Using these data, apparent diffusion coefficient (ADC) maps were calculated on a voxel-by-voxel basis with the software incorporated into the MRI unit. Among the 24 patients without previous treatment, DWI was available in 21 patients.

**Cerebrospinal Fluid Sample Preparation and NMR Spectra**

The frozen cerebrospinal fluid (CSF) samples were thawed and centrifuged at 12,000 rpm for 2 minutes. For nuclear magnetic resonance (NMR) spectroscopic analysis, 360 μL of supernatant was mixed with 90 μL of deuterium oxide (D_2_O) containing a final concentration of 0.025% sodium-3-trimethylsilyl propionate-2,2,3,3-D_4_ (TMSP, Cambridge Isotope Laboratories, Andover, MA) as an internal standard. All NMR spectra were acquired on a 500 MHz NMR spectrometer (Bruker Biospin, Avance 500, Billerica, MA) equipped with a cryogenic probe (Korea Basic Science Institute, Ochang, Korea) [^2^](#_ENREF_2).

**Multivariate statistical analysis**

The processed NMR data sets were imported into SIMCA-P 11.0 (Umetrics AB, Umeå, Sweden), and Pareto scaling was used for mean centering. Both partial least squares-discriminant analysis (PLS-DA) and orthogonal projections to latent structure discriminant analysis (OPLS-DA) analyses were performed using SIMCA-P. The OPLS-DA model was obtained with one predictive (Pp) and two orthogonal components (Po). The OPLS-DA can discriminate groups of interest in the presence of systematic variation. Permutation analysis was used to check the validity and the degree of overfit. The permutation was repeated 100 times. Random forest and Support Vector Machin (SVM) classification were obtained using MetaboAnalyst 5.0. For random forest analysis, the parameters were set as follows, Number of trees: 500, Number of predictors: 7, and Randomness: on. For SVM models, 10-fold cross-validation was selected as the validation method. Diagnostic performance of the multivariate model was estimated by the leave-one-out cross-validation test [^3^](#_ENREF_3). This process was carried out as follows: leaving one of the data out (as a test sample) and building an OPLS-DA prediction model with the remaining data sets. The test sample was considered an unknown sample and predicted by the OPLS-DA model, and the accuracy/reliability of the model was evaluated by comparing with the original value. The above process was repeated for all samples.

**Image Analysis**

MR images were analyzed on a picture archiving and communication system (Infinitt; Infinitt, Seoul, Korea) by two experienced radiologists in consensus. They were blinded to the clinical information and metabolomics profile. MR images were evaluated for lesion multiplicity, extent, contact to the CSF space (subarachnoid space or ventricle), leptomeningeal enhancement, and the ADC value. The lesion extent was evaluated by using the maximum diameter of a tumor on contrast-enhanced T1WI and T2WI. If there were multiple tumors, we measured the maximum diameter for the largest one. One radiologist (J.H.K.; 3 years of brain MRI experience) who was blinded to the patients’ clinical information produced the regions of interest (ROIs) on the ADC map. We calculated the average ADC values for five ROIs, which were drawn over the tumor on the ADC map that matched the enhancing lesion on contrast-enhanced T1WI. Circular ROI diameters ranged from 0.5 to 1 cm.

**Statistical Analysis**

With the simple linear regression model, the CSF protein level and MRI characteristics including the tumor extent based on post-contrast T1WI and T2WI as well as ADC were correlated with marker metabolites. A forward multiple linear stepwise regression analysis was then performed by using nine marker metabolites as independent variables to define models estimating the CSF protein level with a *p* value for entry of 0.05. The Mann-Whitney U test was used to evaluate the differences in the marker metabolite levels between contact to the CSF space (+) vs. (-), leptomeningeal enhancement (+) vs. (-), or multiple lesions vs. a single lesion. Traditional prognostic factors, such as the age, Eastern Cooperative Oncology Group (ECOG) performance status, serum level of lactate dehydrogenase, CSF protein concentration, and involvement of deep regions of the brain (periventricular regions, basal ganglia, brainstem, and cerebellum) were analyzed in a univariate analysis of survival by using the Cox proportional hazard model. Marker metabolites as potential prognostic variables were also examined. A statistical test, the student’s *t*-test, was carried out with OriginPro 8.0 (OriginLab Corporation, Northampton, MA), MedCalc, version 14.8.1 (MedCalc Software, Mariakerke, Belgium), and Excel (Microsoft Corporation, Redmond, WA). All reported *p* values were two-sided, and a *p* value of less than 0.05 was considered to indicate a statistical significance.

**Supplementary Figure**


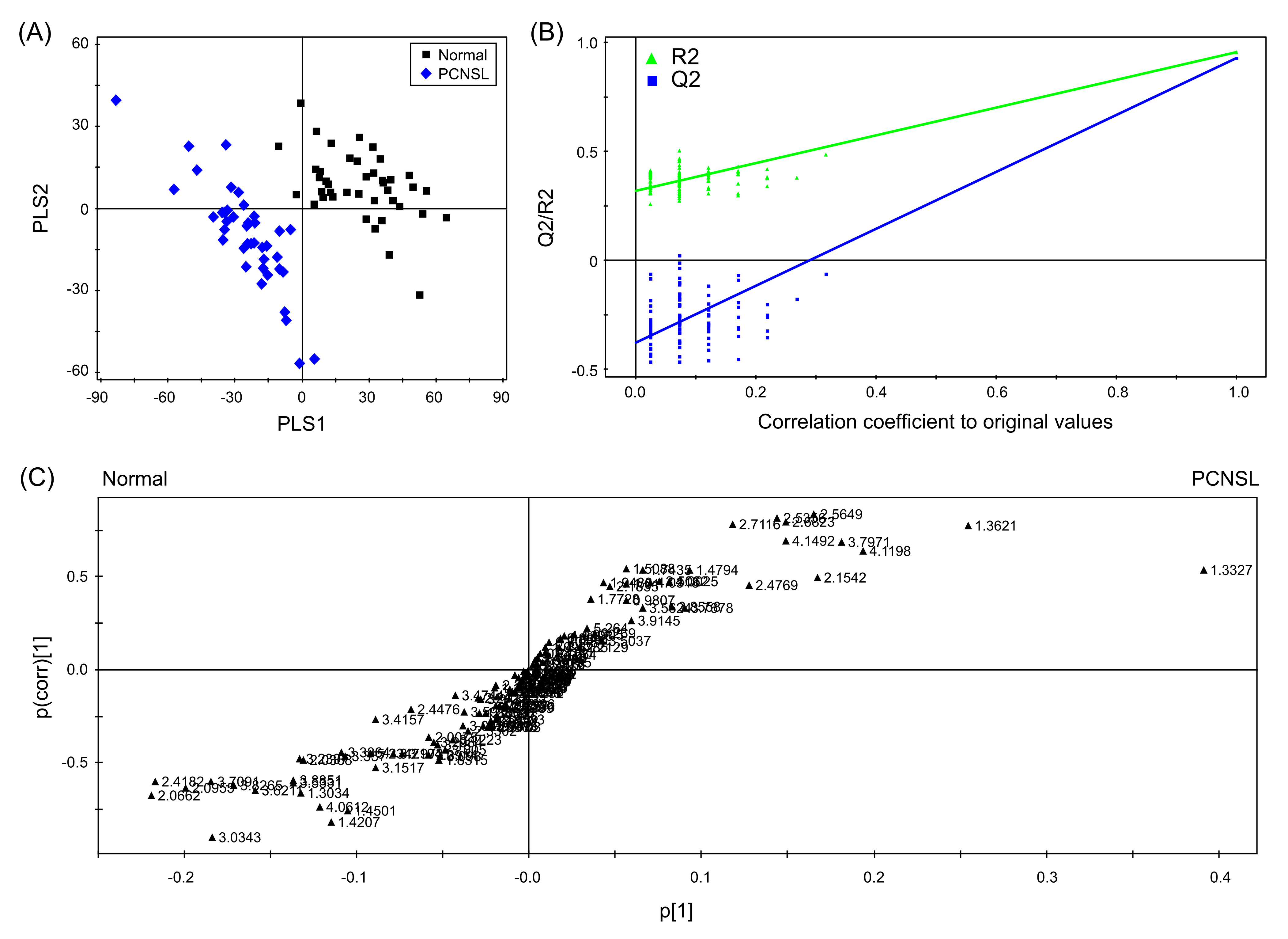


**Supplementary Figure 1.** (A) A partial least square-discriminant analysis (PLS-DA) for the differentiation of PCNSL (blue diamond) and normal (black box) groups. Each symbol represents each participant. (B) Validation model using permutation. For checking the validity, the permutation is repeated 100 times. The blue line and box represent the Q2 value, and the green line and box represent the R2 value. (C) OPLS-DA loadings S-plot showing the variable importance in a model, combining the covariance and the correlation (p(corr)) loading profile. For the statistical analysis, SIMCA-P 11.0 was used.


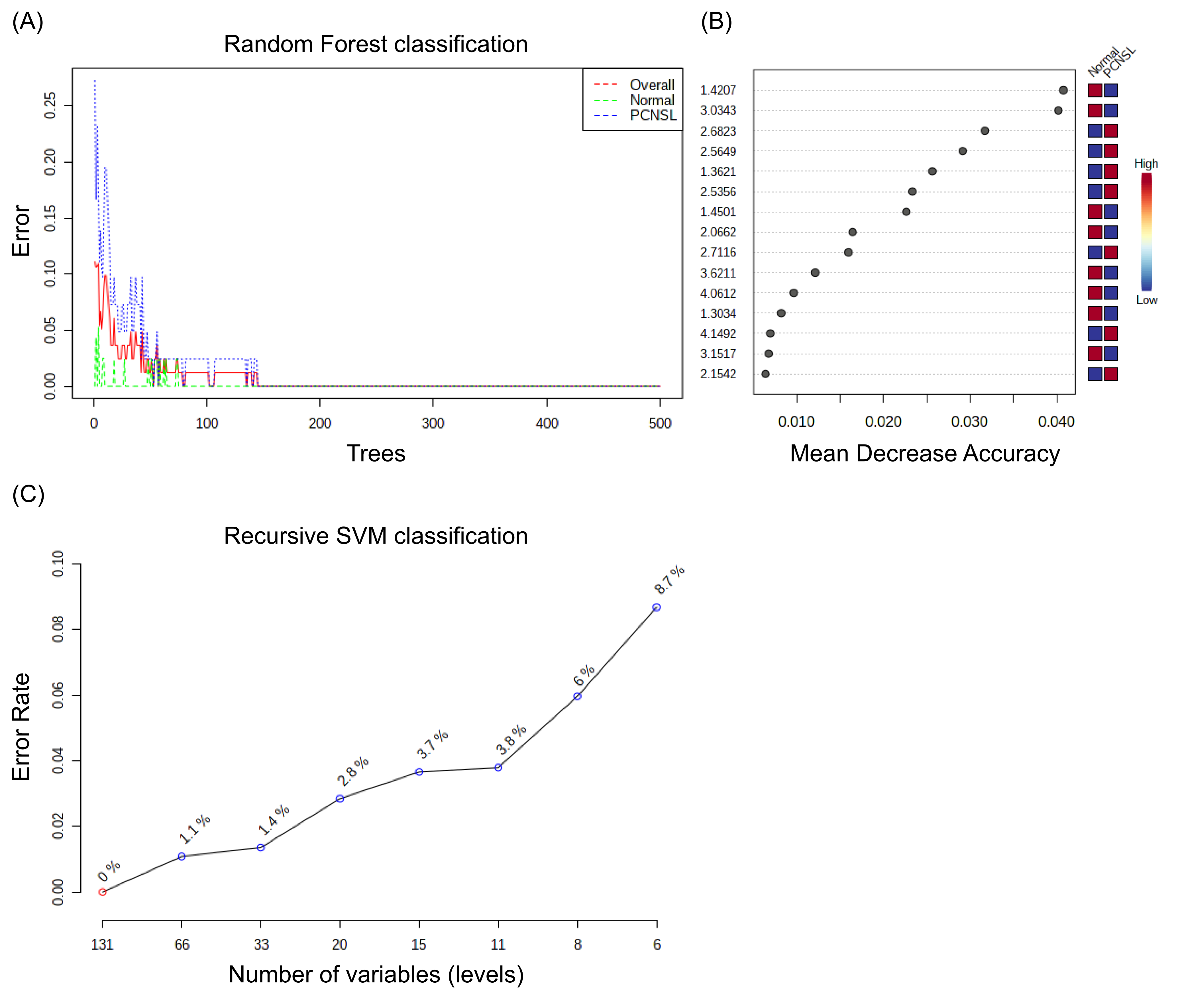


**Supplementary Figure 2.** (A) Cumulative error rates by Random Forest classification. The line showed the error rate of each class. Black line: overall, green line: Normal, blue line: PCNSL. (B) Significant features (^1^H ppm) identified by Random Forest. The features were ranked by the mean decrease in classification accuracy when permuted. (C) Recursive classification with SVM. The best classifier was marked with a red circle. For the statistical analysis, MetaboAnalyst 5.0 was used.

**Supplementary Table 1. Metabolites identified with NMR analysis**

| **Metabolites** | **^1^H (ppm)** | **Fold changes (%)** | | ***p* value** |
| --- | --- | --- | --- | --- |
| 2-hydroxybutyrate | 0.92(d), 1.60–1.75(m), 4.01(m) |  |  |  |
| 3-hydroxybutyrate | 1.15(d), 2.31(m) |  |  |  |
| 3-hydroxyisovalerate | 1.26(s), 2.38(s) |  |  |  |
| Acetate | 1.91(s) |  |  |  |
| Acetoacetate | 2.29(s), 3.45(s) |  |  |  |
| Alanine | 1.47(d), 3.79(q) | ▲ | 60.2 | 2.52 x 10^-6^ |
| Choline | 3.18(s), 3.50(t), 4.08(m) | ▽ | - 63.4 | 4.05 x 10^-4^ |
| Citrate | 2.53(d), 2.68(d) | ▲ | 133.1 | 4.64 x 10^-19^ |
| Creatine | 3.06(s), 3.94(s) | ▽ | - 42.4 | 8.27 x 10^-24^ |
| Glucose | 2.21(t), 3.38-3.91(m), 5.23(d) | ▽ | - 11.4 | 1.25 x 10^-7^ |
| Glutamate | 2.12(m), 2.35(m) |  |  |  |
| Glutamine | 2.09(m), 2.44(m), 3.70(t) | ▽ | -72.3 | 2.87 x 10^-10^ |
| Isoleucine | 0.94(t), 1.02(d) |  |  |  |
| Lactate | 1.33(d), 4.09(q) | ▲ | 25.3 | 6.54 x 10^-6^ |
| Leucine | 0.97(t), 1.72(m) |  |  |  |
| Malonate | 3.15(s) | ▽ | 52.4 | 2.09 x 10^-7^ |
| Mannitol | 3.70-3.92(m) |  |  |  |
| Phosphocholine | 3.21(s), 3.59(m), 4.19(m) |  |  |  |
| Valine | 0.99(d), 1.04(d), 2.27(m) |  |  |  |
| myo-inositol | 3.27(t), 3.56(t), 3.62(t), 4.06(m) | ▽ | - 32.4 | 2.4 x 10^-13^ |

**Supplementary Table 2. Out-of-bag error rate of random forest**

| Randomness | Group | Normal | PCNSL | class error | OOB^*^ error |
| --- | --- | --- | --- | --- | --- |
| On | Normal | 40~41 | 0~1 | 0~0.0244 | 0~0.0122 |
|  | PCNSL | 0 | 41 | 0 |  |
| Use a constant | Normal | 40 | 1 | 0.0244 | 0.0122 |
|  | PCNSL | 0 | 41 | 0 |  |

* OOB error: Out-of-bag error

1. Abrey LE, Batchelor TT, Ferreri AJ, et al. Report of an international workshop to standardize baseline evaluation and response criteria for primary CNS lymphoma. Journal of Clinical Oncology. 2005;23: 5034-5043.

2. An YJ, Cho HR, Kim TM, et al. An NMR metabolomics approach for the diagnosis of leptomeningeal carcinomatosis in lung adenocarcinoma cancer patients. International Journal of Cancer. 2015;136: 162-171.

3. Cho HR, Wen H, Ryu YJ, et al. An NMR metabolomics approach for the diagnosis of leptomeningeal carcinomatosis. Cancer Research. 2012;72: 5179-5187.
